# Modelling metastasis in a dish: *in vitro* approaches for studying breast cancer progression

**DOI:** 10.3389/pore.2025.1612179

**Published:** 2025-09-23

**Authors:** Irem Duman, Verena Pichler

**Affiliations:** ^1^ Department of Pharmaceutical Sciences, University of Vienna, Vienna, Austria; ^2^ Vienna Doctoral School of Pharmaceutical, Nutritional and Sport Sciences, University of Vienna, Vienna, Austria; ^3^ Department of Pharmacy, University of Oslo, Oslo, Norway

**Keywords:** breast cancer metastasis, *in vitro* models, microfluidics, organ-on-chip, cell migration

## Abstract

Cancer metastasis, driven by cell migration, remains the leading cause of cancer-related deaths. In breast cancer, its high metastatic potential underscores the need for better preclinical models to bridge the gap between laboratory findings and clinical outcomes. However, studying migration *in vitro* remains challenging due to the complexity of tumour invasion and the difficulty of replicating physiologically relevant conditions. Traditional two-dimensional (2D) models, such as the scratch assay and transwell migration assay, offer simplicity and reproducibility but fail to capture the tumour microenvironment and dynamic migration behaviours. Advanced three-dimensional (3D) models, including spheroids, organoids, microfluidic systems, and organ-on-a-chip platforms, provide more physiologically relevant conditions but are often limited by cost and technical complexity. This mini-review provides an overview of widely used *in vitro* models for studying breast cancer migration and evaluates their respective advantages, limitations, and future potential. While no single system currently achieves the ideal balance between physiological relevance and practical accessibility, combining complementary tools remains the most effective strategy for investigating the metastatic cascade. Continued innovation in *in vitro* platforms is essential for improving translational accuracy and supporting the development of more effective anti-metastatic therapies.

## Introduction

Cell migration plays a crucial role in biological processes, such as immune responses, wound healing, morphogenesis, and inflammation [1]. In oncology, its significance becomes even more pronounced, as cell migration underpins metastasis the primary driver of cancer-related deaths worldwide. The prognosis for breast cancer patients remains poor, as metastatic disease is typically aggressive and highly infiltrative [2]. Despite its clinical significance, metastasis remains poorly understood and continues to be a major challenge in effective cancer treatment. While large numbers of cancer cells enter blood circulation, studies suggest that less than 0.1% successfully establish secondary tumours [3]. Although only a small fraction of circulating cancer cells successfully metastasize, metastatic disease is responsible for over 90% of cancer-related deaths, highlighting the urgent need to understand the mechanisms governing tumour migration and invasion [4, 5].

Among metastatic cancers, breast cancer has the highest incidences in women worldwide, accounting for 29.4% of cases and 16.7% of cancer-related deaths in Europe (European Commission, October 2023) [6]. Its high mortality rate is largely due to its ability to metastasize to distant organs such as the bone, liver, and lungs. Breast cancer is a heterogeneous disease, classified into molecular subtypes based on tumour receptor status: oestrogen receptor (ER), progesterone receptor (PR), and human epidermal growth factor receptor-2 (HER2). Main molecular subtypes are luminal A (ER/PR-positive), luminal B (ER/PR-positive, higher histological grade), HER2-positive, and triple-negative (ER/PR/HER2-negative). Despite advances in treatment, targeted therapies remain limited for triple-negative breast cancer (TNBC), one of the most aggressive subtypes. While primary breast cancer is highly treatable, no cure exists for metastatic disease [6–10].

**FIGURE 1:**
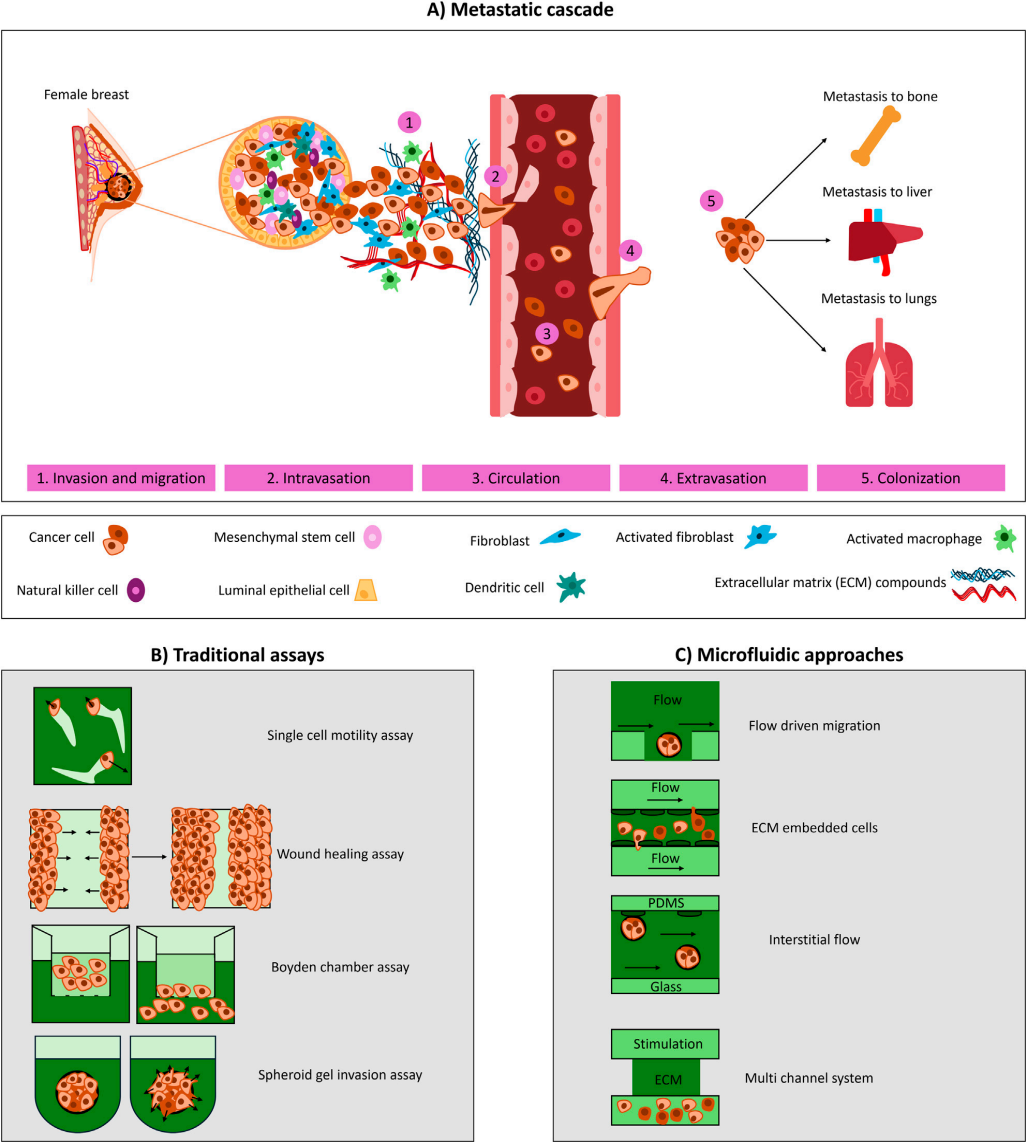
Experimental models to study breast cancer cell migration and metastasis. **(A)** Schematic of the metastatic cascade, outlining invasion/migration, intravasation, circulation, extravasation and colonization. **(B)** Traditional assays used to study migration and invasion: single-cell motility assay (individual cell tracking), wound healing assay (cell migration into a created gap), Boyden chamber (transwell migration across a porous membrane), and spheroid invasion (cell spheroids invading into a surrounding ECM). **(C)** Microfluidic approaches that enable controlled study of migration under flow: flow-driven migration (cells exposed to directional flow), ECM-embedded cultures (cells seeded within an ECM matrix and exposed flow from the sides), interstitial flow (fluid transport across porous matrices), and multichannel systems (parallel channels integrating matrix, stimulation, and culture compartments).

The metastatic cascade involves cancer cell migration from the primary tumour, intravasation into the bloodstream or lymphatic system, circulation, extravasation, and the formation of metastatic colonies [10, 11]. Migration occurs through diverse strategies, including single-cell movement (mesenchymal or amoeboid) and collective migration, influenced by extracellular matrix (ECM) composition, biophysical forces, and tumour microenvironmental conditions like hypoxia and chemoattractants. Once in circulation, cancer cells can exist as individual circulating tumour cells (CTCs) or clusters (circulating tumour microemboli (CTM)), both contributing to metastasis [1, 11–13]. Understanding these mechanisms is essential for developing *in vitro* models that accurately replicate cancer migration dynamics (Figure 1).

Replicating metastasis in preclinical research remains a challenge due to the biological complexity of tumours and patient-to-patient variability [14, 15]. Traditional pre-clinical studies have largely relied on two-dimensional (2D) *in vitro* models to evaluate drug efficacy, but these fail to replicate the three-dimensional (3D) tumour microenvironment, leading to discrepancies between pre-clinical and clinical findings. Consequently, approximately 95% of cancer drugs effective in preclinical trials fail in clinical settings, with only 7.5% progressing beyond Phase 1 trials. This highlights the urgent need for physiologically relevant models that better capture cancer progression, particularly in the context of metastasis and migration [16].

This review examines *in vitro* tools for studying breast cancer migration, focusing on models that replicate different steps of the metastatic cascade. Their limitations, missing aspects, and potential improvements will be discussed briefly. As preclinical models evolve, they hold promise for uncovering drug resistance mechanisms and contributing to the development of more effective treatment strategies.

## Overview of tools for studying metastasis

### *In vitro* tools for studying invasion and migration

The first step in metastasis is cancer cell invasion and migration, initiated by penetration of the basement membrane and movement through the ECM, either individually or collectively. Malignant transformation facilitates ECM degradation, driven by collagen deposition, fibre thickening, and linearization, resulting in increased stiffness [17]. Accurate *in vitro* models must replicate ECM properties such as stiffness, pore size, and degradability [18]. Scratch and transwell assays are commonly used to study breast cancer migration and invasion, each offering specific strengths and limitations (Table 1).

**TABLE 1:** Summary of commonly used *in vitro* assays for studying breast cancer cell migration and invasion. Each assay offers distinct advantages and limitations, particularly in terms of physiological relevance, imaging capability, and ability to replicate tumour microenvironmental conditions.

Assay	Description	Advantages	Limitations/research gap
Scratch (Wound Healing) Assay [5, 19, 20]	Measures 2D cell migration by creating a “wound” in cell monolayers and observing gap closure time	Simple, cost effective and real time visualization possible	Lacks 3D ECM interactions; manual scratching introduces variability; cannot replicate *in vivo* conditions; impact of tumour microenvironment factors on migration dynamics
Transwell Migration/Invasion Assay [18, 21]	Cells migrate through a porous membrane toward chemoattractant. Invasion: the membrane is coated with ECM (e.g., Matrigel). Intravasation: monolayer of endothelial cells (e.g., HUVECs) is cultured on a porous membrane, and breast cancer cells are seeded on top	Quantifies chemotaxis and invasion; allows comparison of migration rates between cell types	No real-time visualization; oversimplifies cell-ECM interactions; lacks dynamic microenvironmental conditions; influence of shear stress and flow dynamics on invasion; integration of immune or stromal cell interactions
Spheroid Invasion Assay [16, 22, 23]	Tumours spheroids are embedded in ECM-like hydrogels to study radial outgrowth of invasive cells into the surrounding matrix	Mimics *in vivo* tumour invasion; reveals differences between aggressive and less invasive subtypes	Variability in spheroid size and matrix density effects reproducibility; requires advanced imaging quantification; mechanism driving CTC cluster formation during spheroid invasion; patient specific variability in response
Microfluidic Models [24, 25]	Lab-on-a-chip devices simulate vascular structures, enabling real-time visualization of cancer cell intravasation or circulation under shear stress conditions	High physiological relevance; replicates flow dynamics and co-culture with stromal or immune cells	Expensive, technically complex, limited accessibility for many labs; standardization of protocols, scalability for high-throughput drug screening

Wound healing (scratch) and transwell assays allow for basic evaluation of cancer cell motility. The scratch assay involves creating a controlled gap or ‘wound’ in a confluent monolayer, typically using a pipette tip, and monitoring its closure over time to assess migration. While those assays are informative, its 2D nature and lack of correct ECM interaction limit physiological relevance.

As example, one study investigated the effects of lidocaine, a local anaesthetic, on breast cancer cell migration by examining its interaction with the TRPM7 ion channel which regulates ion flow and has been linked to cancer cell migration and mechanotransduction. Using six different breast cancer cell lines, including knockout models, the researchers evaluated viability and migration through 3-(4,5-Dimethylthiazol-2-yl)-2,5-Diphenyltetrazolium Bromide (MTT), wound healing, and electrophysiological assays. The findings revealed variable responses depending on cell type and dosage, contributing to a better understanding of lidocaine’s clinical effects Yet, the inability of the scratch assay to model tumour microenvironment dynamics, such as ECM stiffness and structural cues, leaves key mechanistic questions unresolved [26].

To enhance relevance, researchers may coat surfaces with ECM components or use the ring assay, which confines cells within a barrier on an ECM-coated surface. This format improves reproducibility by standardizing wound size and cell distribution. Yet, even these modified 2D methods lack the spatial complexity of 3D environments where cells interact with ECM in all dimensions [21].

The transwell assay assesses chemotaxis, invasion, and migration through porous membranes and can be ECM-coated (e.g., fibronectin) to mimic specific matrix components (Table 1). A recent study combined transwell and scratch assays to show that MitoQ, a mitochondria-targeted Reactive Oxygen Species (ROS) inhibitor in Phase I trials, effectively suppressed breast cancer cell migration, highlighting its potential as an early anti-metastatic agent [27].

However, these 2D models lack critical features of the 3D tumour microenvironment, such as complex cell–matrix interactions and diverse migration modes. The scratch assay misses dynamic and collective behaviours, while the transwell model, despite ECM coating, cannot replicate native ECM architecture, shear stress, or real-time dynamics. Key factors like ECM stiffness, structure, and immune–stromal interactions remain underrepresented [18, 27].

To address limitations of 2D models, 3D spheroid invasion assays have become widely used. Breast cancer cells (e.g., MDA-MB-231, SUM149, T47D) are cultured as spheroids and embedded in ECM-like hydrogels (e.g., collagen, Matrigel), enabling radial invasion (Table 1) [28]. This approach better mimics *in vivo* tumour invasion, particularly in highly invasive TNBC. Spheroid assays distinguish between invasive and less invasive subtypes, with mesenchymal-like TNBC cells showing greater invasiveness than luminal types [29]. They also allow investigation of tumour cell interactions. For example, one study showed that invasive 4T1 cells promoted the migration of non-invasive 67NR cells through matrix degradation and invadopodia formation. However, the study also highlighted model limitations such as the absence of mechanical cues, limited pathway analysis, and a lack of immune or fluidic components [30]. Additional challenges include variability in spheroid size and matrix density, as well as imaging difficulties that impact reproducibility. To address these, spheroid assays are being combined with microfluidic platforms, which offer controlled flow, co-culture options, and real-time imaging of invasion under dynamic conditions [31].

In summary, while 2D assays like scratch and transwell remain foundational tools for studying cancer cell migration, their inherent limitations underscore the need for more physiologically relevant models. 3D spheroid and microfluidic systems provide a closer approximation of the tumour microenvironment and enable more accurate investigation of invasion dynamics, offering a valuable path forward in metastasis research.

### *In vitro* tools for studying intravasation

Intravasation, the second step of metastasis, involves cancer cells crossing endothelial barriers to enter the bloodstream or lymphatic system [32]. It is driven by molecular changes that enhance interactions with vascular structures, influenced by the tumour microenvironment. This environment includes endothelial cells, fibroblasts, lymphocytes, extracellular vesicles, and signalling molecules that collectively support angiogenesis, immune modulation, and ECM remodelling, all contributing to intravasation [33, 34].

To model this process, various *in vitro* and microphysiological systems assess proliferation, migration, vessel formation, and endothelial barrier function. A commonly used method is the modified transwell assay, which offers a simple and cost-effective platform for studying cancer cell movement across an endothelial layer [35]. However, it lacks key *in vivo* features such as flow dynamics, 3D architecture, and interactions with immune or stromal components. These limitations have prompted the development of microfluidic technologies, which offer more structurally and dynamically accurate models of intravasation. These advanced platforms incorporate spheroids, co-cultures, and organoids within endothelial-lined vessels, allowing real-time visualization of cancer cell entry under flow and shear stress [36]. They also enable co-culture with stromal or immune cells in defined ECM conditions. For instance, Cho et al. [37], developed a dual-channel microfluidic model mimicking blood and lymphatic vessels to study EMT-induced angiogenesis, revealing how inflammatory cytokines drive vascular remodelling and invasion [37].

Despite these advances, microfluidic systems remain limited by cost, technical demands, and challenges in modelling long-term interactions and immune responses. Future progress depends on developing standardized, accessible platforms that integrate patient-derived organoids and key tumour microenvironment components to enhance clinical relevance.

#### *In vitro* tools for studying circulation

The third step of metastasis is circulation. Only a small fraction of cancer cells enters the bloodstream, and even fewer survive the hemodynamic shear forces, immune defences, and interactions with red blood cells. CTCs, which can be single cells or clusters, exhibit higher metastatic potential when in clusters. Microfluidic technologies have advanced the study of CTCs by isolating them from patient blood samples using functionalized surfaces coated with adhesion proteins or antibodies, improving our understanding of metastatic potential and disease progression [38].

These platforms also enable the study of mechanical forces affecting CTC behaviour, such as fluid shear stress, which influences survival, deformation, and motility, key factors in secondary tumour formation. It’s important to also consider the lymphatic system, where lower shear stress may promote different migratory behaviours and survival strategies. Simulating both blood and lymphatic circulation conditions in microfluidic models enhances physiological relevance [39, 40].

One study developed a microfluidic platform to explore interactions between circulating immune cells and micro metastases, with potential applications in immunotherapy [41]. Despite their promise, microfluidic systems face challenges like technical complexity, high costs, lack of standardization, and low throughput, limiting their adoption and large-scale screening use.

In summary, microfluidic platforms have advanced CTC research, but improving model accessibility, throughput, and physiological relevance is necessary to fully capture the complexity of circulation in metastasis.

#### *In vitro* tools for extravasation and colonization

The final step of the metastatic cascade is extravasation and colonization. After surviving in circulation, cancer cells must establish themselves at new sites. Unlike intravasation, where cells migrate toward leaky, tumour-modified vasculature under chemotactic and durotactic gradients, extravasation occurs through healthier blood vessels. Here, cancer cells experience fluid shear stresses due to blood flow as they breach the vasculature and invade surrounding tissues [33].

Transwell-based systems have been used to study extravasation by assessing cancer cell migration toward chemoattractants like fetal bovine serum (FBS) [35]. While useful for basic motility studies, they lack plasma proteins, blood cells, and flow dynamics, limiting physiological relevance. FBS does not mimic the rheological properties of blood, and static systems cannot replicate *in vivo* vascular cues. Therefore, *in vivo* tail-vein injection models are often used for their physiological relevance, introducing cancer cells into circulation via the lateral tail vein [5, 42, 43]. However, these models cannot capture early metastatic steps like invasion or intravasation and pose challenges for real-time imaging. The portal vein-liver injection model was established to study breast metastatic colonization. In this approach, tumour cells are injected into the portal vein, from where they inoculate the liver and can further enter the circulation [44, 45]. While this method achieves efficient colonization and allows controlled tumour cell delivery, it does not capture the full metastatic cascade, particularly the early steps from a primary tumour, and instead models only the later stages of colonization and outgrowth.

To bridge these gaps, advanced *in vitro* systems have been developed. Specially microfluidic platforms address these issue by enabling co-culture of endothelial, stromal, and cancer cells, incorporation of flow, and real-time imaging under dynamic conditions, making them state-of-the-art for physiologically relevant modelling [46]. For instance, Jeon et al. [46] developed a microfluidic model with breast cancer and endothelial cells in a collagen matrix for high-resolution imaging of extravasation [46]. To model bone metastasis, another study used a decellularized bone matrix instead of soft hydrogels and incorporated interstitial flow and co-culture with bone marrow-derived mesenchymal stem cells, better mimicking breast cancer colonization in bone [8].

To better model breast cancer extravasation into common metastatic sites such as the lung and bone, Kwak and Lee [47] developed a vascularized tumour-on-a-chip system [47]. They engineered blood vessels by culturing human microvascular endothelial cells and embedded parenchymal organ cells in a surrounding gel to mimic the ECM of target organs. Breast cancer cells with specific metastatic properties were then introduced into the engineered vessels to investigate their extravasation into distant organs, particularly bone. Their findings revealed that osteoblasts play a crucial role in the selective extravasation of bone-seeking cancer cells. This study underscores the potential of vascularized tumour-on-a-chip models for replicating metastasis and extravasation in distinct organ microenvironments.

In a separate study, the role of monocytes in tumour cell extravasation and the metastatic cascade was investigated using a vascularized microfluidic platform. Human fibroblasts and endothelial cells were cultured to form microvascular structures within a fibrin hydrogel, alongside breast cancer cells. The findings demonstrated that monocytes could reduce cancer cell extravasation in a non-contact-dependent manner. However, once monocytes transmigrated through the vasculature and differentiated into macrophage-like cells, their effect on cancer cell extravasation was minimal [48].

While 3D models and microfluidic platforms have enabled the *in vitro* replication of key components of the tumour microenvironment, most still lack a fully perfusable vascular network, a critical feature for accurately modelling both intravasation and extravasation [49]. Looking ahead, integrating immune components, patient-derived matrices, and dynamic vasculature into *in vitro* systems will be essential to more faithfully capture the complexity of breast cancer extravasation and colonization.

## Advanced models and future directions

Various *in vitro* models have been developed to mimic different stages of the metastatic cascade, each with its own strengths and limitations. This mini-review summarizes the most commonly used methods, highlights recent advancements, and discusses their challenges. Cancer cell migration can be likened to an obstacle race, where each step presents unique biological and biochemical challenges. Researchers aim to replicate these complexities *in vitro*, integrating as many relevant factors as possible to develop more physiologically relevant platforms.

A comprehensive understanding of the metastatic cascade is crucial for designing effective antimigratory strategies. However, addressing metastasis alone is not enough, cancer progression is further complicated by challenges that arise during and after treatment. Advanced methods combine different organ models on one chip, to address these complications. One notable example addressing such complexities is the heart–breast cancer-on-chip model developed by Lee et al. [50] to monitor chemotherapy-induced cardiotoxicity [50]. This platform, utilizing induced pluripotent stem cells, represents a significant advancement in personalized treatment approaches. However, its major drawback is accessibility.

The MIRO (Micro Immune Response On-chip) platform is a promising model that mimics the spatial organization of HER2+ breast cancer cells, stromal barriers, and immune cells in a 2.5D humanized co-culture system [51]. It enables real-time tracking of immune cell motility and highlights the role of stromal barriers in immune exclusion, a key resistance mechanism. MIRO improves on traditional models by eliminating non-human components (e.g., rat collagen) and being compatible with multiwell plates for parallel drug testing. However, its complex microfluidic design limits scalability, and it cannot fully replicate tumour features such as vascularization and neuro-immune interactions. Despite these challenges, MIRO contributes to personalized medicine by offering insights into metastasis and treatment resistance.

Two *in vivo* models, the tail vein or portal vein injection models, are described in literature for breast cancer. The former is more broadly applied potentially due its simplicity compared to the portal vein injection [42–45].

Another recent advancement is the integration of AI and machine learning into cancer model systems. The Prediction Wound Progression Framework (PWPF), a deep learning (DL) approach, enhances wound healing assay analysis by predicting breast cancer cell migration dynamics [52]. By combining synthetic and experimental data, PWPF improves the scalability and precision of 2D migration studies, bridging traditional assays and computational modelling. It shows that DL models, trained on artificial data and fine-tuned with real images, can effectively predict migration patterns. Technical barriers remain, including variability in experimental conditions and the high computational demand of training DL models like Convolutional Long Short-Term Memory (ConvLSTM). In another study, Yadav et al. [53] developed machine learning tools to convert single-cell behavioural data into population-level survival predictions [53].

Looking forward, AI tools could also be used to segment and quantify cell migration in high-content imaging workflows, analyse cytoskeletal dynamics at single-cell resolution, or even guide adaptive experimentation in real time. As *in vitro* models become increasingly complex, integrating machine learning will be essential for efficiently extracting actionable data from large-scale experiments.

## Conclusion

Traditional *in vitro* assays like the scratch and 3D invasion assays remain widely used due to their simplicity, affordability, and ease of use, despite limited physiological relevance. In contrast, microfluidic and organ-on-a-chip systems offer more complex, realistic models but are costlier and technically demanding.

Effective cancer migration research requires balancing accessibility with model complexity. Simpler assays are valuable for rapid, reproducible insights, while advanced platforms enable detailed mechanistic studies. Model selection should align with the specific research question, integrating both basic and sophisticated systems to enhance understanding and therapeutic development.

Looking ahead, *in vitro* models will continue to evolve, better mimicking key steps of metastasis and reducing reliance on animal testing. Strategic model selection is essential for translational relevance. Importantly, the literature often emphasizes successful results, overlooking the time, resources, and animal use behind failed experiments. This highlights the need for a deliberate, critical approach to selecting and refining *in vitro* systems, ensuring they bridge the gap between lab findings and clinical outcomes and drive both fundamental and translational cancer research.

## Abbreviations

2D, Two dimensional; 3D, Three dimensional; 4T1, Mouse triple negative breast cancer cell line; 67NR, Mouse breast cancer cell line; ConvLSTM, Convolutional Long Short-Term Memory; CTC, Circulating tumour cells; CTM, Circulating tumour microemboli; DL, Deep learning; ECM, Extracellular matrix; ER, Oestrogen receptor; FBS, Fetal bovine serum; HER2, Human epidermal growth factor receptor-2; MDA-MB-231, Human triple negative breast cancer cell line; MIRO, Micro Immune Response On-chip; MTT, 3-(4,5-Dimethylthiazol-2-yl)-2,5-Diphenyltetrazolium Bromide; PR, Progesterone receptor; PWPF, Prediction wound progression Framework; ROS, Reactive Oxygen Species; SUM149, Human triple negative breast cancer cell line; T47D, Human luminal A subtype breast cancer cell line; TNBC, Triple-negative breast cancer.
